# Evaluation of the health information system for monitoring and evaluating the voluntary medical male circumcision program in Mozambique, 2013-2019

**DOI:** 10.11604/pamj.2022.42.236.28534

**Published:** 2022-07-27

**Authors:** Hélio Inácio Elias, José Carlos Langa, Judite Monteiro Braga, Zandile Nukeri, Erika Valeska Rossetto, Cynthia Sema Baltazar

**Affiliations:** 1 Mozambique Field Epidemiology Training Program, National Institute of Health, Maputo, Mozambique,; 2 Faculty of Medicine, Eduardo Mondlane University, Maputo, Mozambique,; 3 National Institute of Health, Maputo, Mozambique,; 4 MassGenics, Assigned to Mozambique Centers for Disease Control and Prevention, Maputo, Mozambique

**Keywords:** Health information systems, evaluation, VMMC, HIV, Mozambique

## Abstract

**Introduction:**

the prevalence of human immunodeficiency virus (HIV) in Mozambique has increased from 11.5% in 2009 to 13.2% in 2015. The Mozambique Ministry of Health (MOH) developed a 5-year strategy (2013-2017) for male voluntary medical circumcision (VMMC) to increase in the provinces where there is the greatest number of HIV. We aimed to evaluate the health information system for monitoring and evaluating VMMC in Mozambique from 2013-2019.

**Methods:**

we reviewed the records of the National Health Information System for Monitoring and Evaluation (SIS-MA) database for VMMC of the MOH. The evaluation was based on the updated guidelines for the evaluation of public health surveillance systems of the Centers for Disease Control and Prevention.

**Results:**

the coverage rate for VMMC in Mozambique in the period under study was (89%) (1,784,335/2,000,000). The system was expected to circumcise for the year 2019 (162,052) and 390,590 was reached, exceeding the target 241.0% (390,590/162,052). Of the total number of men circumcised, 0.7% (12,391/1,784,335) were HIV-positive (previously tested) and 0.4% (6,382/1,784,335) had a record of adverse events in the period under review (2013-2019). Zambézia Province had the highest VMMC coverage (in numbers) at 16.0% (396,876/2,476,395) while Maputo City had the least 19.7% (107,104/543,096). The system was able to operate both online and offline and continue functioning with introducing new changes (e.g. the new male circumcision complication reporting).

**Conclusion:**

the system was representative, flexible, simple, with good data quality and low acceptability. We recommended continuous and routine entry of quality data into the system, guide organizations for improved functioning.

## Introduction

Voluntary medical male circumcision (VMMC) is a preventive procedure that reduces the risk of contracting HIV in men through heterosexual transmission. It is an essential component of the World Health Organization (WHO) and Joint United Nations Programme on HIV/AIDS (UNAIDS) strategy to end Acquired Immunodeficiency Syndrome (AIDS) by 2030 and has been implemented in the countries of Southern and Eastern Africa highly affected by HIV/AIDS [1].

The purpose of the evaluation of the public health surveillance systems is to ensure that the problems of importance to public health are being monitored in an efficient and effective manner, knowing that HIV/AIDS is a public health problem in the country, the ministry of health adopted control measures in accordance with the recommendations that emerged after several studies carried out in the region on the protective factor of male circumcision as another method of prevention against HIV [2,3]. Approximately 30% of men aged 15 and over, have been circumcised worldwide [4]. Southern African countries with high HIV prevalence, such as Botswana, Lesotho, Malawi, Mozambique, Namibia, South Africa, Swaziland, Zambia and Zimbabwe, have a relatively low prevalence of male circumcision, of these countries, Zimbabwe recorded the lowest prevalence of male circumcision with 10.3% [5].

However, there are countries in the same region where more than 80% of men have been circumcised and have low HIV/AIDS prevalence rates, such as Angola with 3.7%, and Madagascar with 0.5% [6]. UNAIDS and the United States President's Emergency Plan for AIDS Relief (PEPFAR) have estimated that increasing VMMC in men aged 15-49 years old in 14 southern and eastern African countries will require 20.3 million circumcisions every five years to reach 80% coverage of the eligible population [7].

Using this level of coverage over the next 15 years, mathematical modelling suggested that there is potential to avoid up to 3.4 million or 22% of new HIV infections by 2025 [3,7]. Between 2016 and 2025 an additional 8.4 million circumcisions would be required to maintain the 80% level of coverage and a potential savings of 16.5 billion would be generated through avoided costs in HIV care and treatment [3,7,8]. In Mozambique, nearly 2.2 million people are living with HIV/AIDS, with nearly 297 new infections and 170 deaths per day [9-11]. Although the prevalence of HIV in Mozambique has increased from 11.5% in 2009 to 13.2% in 2015, the prevalence of male circumcision hasn´t reached the desired target of 2 million males circumcised per year and 48.6% of the national goal had been achieved as of 2017 [1,6,11].

There is growing scientific evidence that VMMC is a cost-effective intervention to reduce the risk of transmission of HIV transmission and other sexually transmitted infections by 50-60%, in combination with other strategies [3,7,8,11]. The Mozambique Ministry of Health (MOH) developed a National Male Circumcision Strategy (NMCS) for 2013-2019 which aimed to perform VMMC (representing nearly 80% coverage) among men aged 15-49 in the provinces with the highest HIV prevalence (Maputo City, Maputo Province, Gaza, Manica, Sofala, Tete, and Zambézia) [6,11].

In Mozambique, the VMMC service is largely provided in urban health facilities and is supported by health partner organizations. The program uses standardized tools for data management and reporting from the health facility level to the national level. The system is both paper and electronic based. The purpose of evaluating public health surveillance systems is to ensure that problems of public health importance are being monitored in an efficient and effective manner and to provide key information for strategic decision making. In Mozambique, there are limited studies conducted to evaluate the SIS-MA on the platform of male circumcision. We conducted an evaluation of the health information system for monitoring and evaluation (SIS-MA) for VMMC in Mozambique to assess the performance of the system and its importance for the public health of Mozambique. The results can support the building and scaling of a more robust system, improve service, and advocate for more integrated VMMC services.

## Methods

**Study design:** a database of voluntary medical male circumcision programs in Mozambique was considered where data from the period 2013-2019 were extracted. A descriptive cross-sectional evaluation was conducted based on the updated guidelines for evaluating public health surveillance systems from Centers for Disease Control and Prevention (CDC) [2]. Voluntary medical male circumcision data collected from 2013 to 2019 was extracted from SIS-MA. Evaluation parameters were used (Table 1, Table 1 suite), and data from National Statistical Institute (INE), 2017 (IV Mozambican population census) were reviewed to calculate VMMC coverage in the period (2013-2019) [9,10-11].

**Table 1:** evaluation of the health information system for monitoring and evaluating the voluntary medical male circumcision program in Mozambique, 2013 -2019

Attribute	Rating criteria	Parameter	Values achieved	Punctuation
Simplicity	Existing variables	≤50 variables=1	≤ 30 variables=1	0 to 4 points
	Number of organizations involved	˃50 variables=0	7 organizations=0	Rating <3= complex
	Information submission levels	≤4 organizations=1	4 levels=1	≥ 3 = simples
	Transmission of information	>4 organizations=0	Online=1	Achieved: 3/4
	Offline (telephone, internet, and SMS) or online	≤4 levels=1		
		>4 levels =0		
		Offline=1		
		Online=0		Classification: simples
Acceptability	Proportion of reports sent on time	< annual target = 0	10,128 reports=0	0 to 2 points
	Follow-up of individuals VMMC adverse event	≥ annual target = 1	4652 reports =1	0 = not acceptable
		Lack of follow-up=0; follow up completed =1		1 = low acceptability
				2 = acceptable
				Achieved:1/2
				Classification: 1 = low acceptability
Flexibility	Ability to adapt to changes, when introducing new variables	No= 0	Yes=1	0 to 1 points
		Yes= 1		0 = not flexible
				1 = flexible
				Achieved:1/1
				Classification: flexible
Data quality	Mandatory and essential fields filled in the SIS-MA research form: age	0-2 fields; filled = bad		0 to 6 points
		3-4 field; filled=regular		0 = bad
		≥5-7 high	7 fields filled=1	1 = low
	Clinical procedures	0-2 fields filled = bad		2 = regular
		3-4 field filled=regular		3 ≥ high
		≥5-7 high	7 fields filled=1	Achieved:6/6
	Serological status	0-2 fields filled = bad		
		3-4 field filled=regular		
		≥5-7 high	7 fields filled=1	

**Table 1 suite:** evaluation of the health information system for monitoring and evaluating the voluntary medical male circumcision program in Mozambique, 2013 -2019

Attribute	Rating criteria	Parameter	Values achieved	Punctuation
Data quality	Follow-up	0-2 fields filled = bad		
		3-4 field filled=regular		
		≥5-7 high	7 Fields filled=1	
	Adverse event VMMC)	0-2 fields filled = bad		
		3-4 field filled=regular		
		≥5-7 High	7 Fields filled=1	
	Data consistence (concordance) of SIS-MA and report	< 50 = 0		
		51-70= 1		
		70 to 89%= 2		
		High ≥ 90%=3	≥ 90%=1	Classification: 1 = high data quality
Stability	Ability to remain operational (frequency of interruptions due to lack of personnel and material)	With interruption=0	Without interruption = 1	0 to 1 points
				0 = not stality
				1 = stalility
				Achieved:1/1
		Without interruption=1		Classification: stability
Timeliness/Opportunity	Time required to accomplish all steps of the system (from data collection at health facility, to the analysis and retro information at central level - MISAU)	> 48 hours = 0	≤48 hours = 1	<1= not opportune
	For circumcision resulted in complicated	≤ 48 hours = 1	≤ 48 hours = 1	2= regular
	Up to 30 days for general information of procedure	>48h = 0		3 = opportune
		≤ 48h = 1		Achieved: 3/3
		>30 days = 0		
		≤30 days = 1		
		Time trend: 100%	≤ 48 hours = 1	Classification: opportunity
Representativeness	The ability to accurately describe the by time (data of procedure), person (age and clinical status) and place (province district, health facility)	Description of cases by person: 85%	<85=not representative	<85=not representative
		Place: 85%	≥85%=representative=1	≥85%=representative
				Achieved: 3/3
				Classification: representative

**Setting:** a descriptive cross-sectional, VMMC data collected from 2013 to 2019 was extracted from SIS-MA.

**Participants:** although it did not involve the selection of participants directly, eligibility criteria for this study were all men registered by the system in the period under review 2013-2019. It did not involve the selection of participants as such. It was a secondary study where it was possible from reports and databases. Although it did not involve the selection of participants directly, the eligibility criteria for this study were all men registered by the system in the period under review 2013-2019. It did not involve the selection of participants as such. It was a secondary study where it was possible from reports and databases.

**Variables:** this study focused on system evaluation and the number of monthly VMMC report forms on different discrete attributes was analysed, number of organizations involved in the system; reporting levels; number of reports expected; number of existing variables; number of procedures performed during the reviewed study period; the following attributes were evaluated from the CDC 2001 guideline: simplicity, acceptability, flexibility, data quality, stability, timeliness, and representativeness.

**Data sources/measurement:** a descriptive analysis of the data was performed using the Microsoft Excel 2019 and, as secondary data exist in the National Tobacco Control Programme (NTCP), ethical approval was not required as identified datasets were used. To minimize bias, data cleansing was performed.

**Study size:** a descriptive cross-sectional evaluation was conducted based on the updated guidelines for evaluating public health surveillance systems from Centers for Disease Control and Prevention.

**Quantitative variables:** simplicity: verification of existing form; 1) VMMC monthly report form; number of organizations involved in the system; reporting notification levels; acceptability: number of expected reports; flexibility: the system's ability to adapt to different realities and changes in the scope of the need to respond to a public health event in the program was verified. Data Quality: 30 essential variables that must be entered were checked, giving a score of 4/4 for data quality. The completeness of essential variables such as procedures, HIV status, follow-up information, and complication of circumcision was analyzed. As for consistency, the concordance of the data reported by SIS-MA was calculated, acceptable if ≥90%, and the deviation of the data was checked. Stability: this attribute was evaluated using the frequency of interruptions in system operation due to staff shortages. Timeliness: steps in the process of transmitting information from one level to the next and representativeness: number of procedures performed during the revised study period.

**Statistical methods:** we analyzed the data using Excel 2019, for the presentation of the data we used tables. The male circumcision program at public health directorate, MOH, gave permission to conduct this evaluation.

## Results

**Description of the system:** VMMC was implemented in March 2009 in small surgery services under an integrated perspective for HIV control strategy. Post-operative medical checks were scheduled after VMMC on day 2, 7 and 42. Key strategies to ensure the safety of procedures and minimize the risk of adverse events were implemented. Those strategies included implementation of quality assurance measures, assurance of health provider competency, and monitoring continuous quality improvement [8,11]. In accordance with the five-year scale up strategy, the VMMC program was separated from the broader HIV program (also referred has HAP).

**Figure 1:**
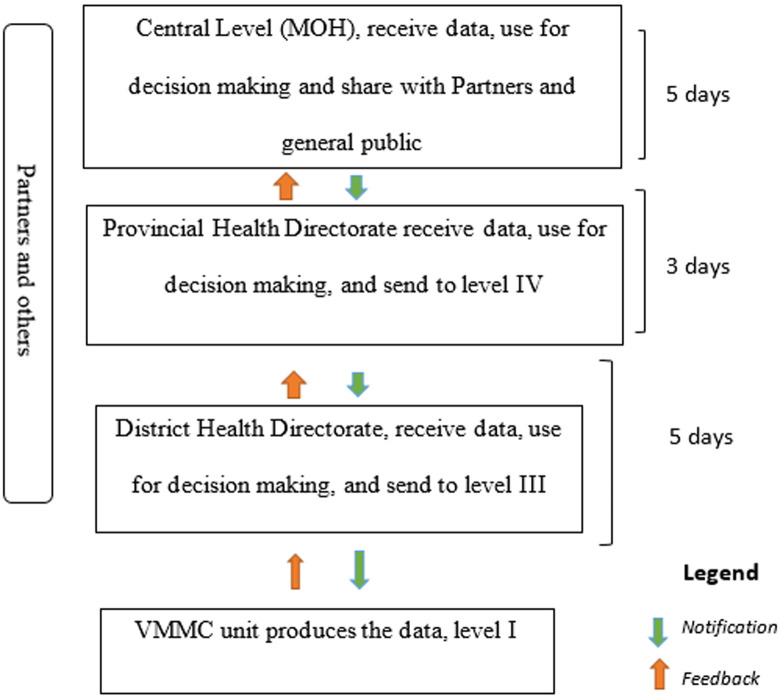
flow of information flow system of the VMMC program in Mozambique, from 2013-2019

The VMMC data was integrated into SIS-MA in 2013. In the same year, the program developed, piloted and introduced new registration and notification forms that started to be used in the health facilities where male circumcision was performed. The SIS-MA system enabled the collection, analysis, interpretation and provided systematic dissemination of aggregate health district data to the country's central level (Figure 1). Retrospective data entry typically took less than 90 days and less than 48 hours to report adverse VMMC events. Typically took less than 90 days and less than 48 hours for the reporting of VMMC adverse events. The system had 4 levels of information (health facility, district, province, and national) and more than 4 organizations (includes: CDC, WHO, I-TECH, USAID, Doctors without Borders, Jhpiego, ICAP, PSI, ICAP) were involved in the information system.

The MOH, with partner organizations´ support, planned for a rapid scale up of VMMC, which required health system strengthening. This included health staff training, improvement in health facility infrastructure, and commodities and equipment procurement. In 2013 the coverage rate for VMMC in the country was 37.0% (146,046/146,046,37), and in 2019 it increased to 241.9% (391,989/162,052), as more procedures were carried out than the expected target (162,052). By the end of 2017, 46 health facilities for VMMC, 140 temporary health facilities for VMMC and 16 mobile units (to provide VMMC services in locations with limited or no access to health care services or infrastructure) were implemented under the NMCS. To ensure adherence to international quality standards, ensure safety, and comply with ministry of health clinical guidelines, all VMMC clinics participated in an external quality assurance (EQA) process [8,11].

### Description of attributes

**Simplicity:** we found that the circumcision report form had 30 variables, and there were 3 forms to be completed in the system: 1) the VMMC monthly report form; 2) VMMC client record form; and (3) VMMC adverse event notification form. There were more than 4 organizations supports and participate on the system: USAID, the Centers for Disease Control and Prevention (CDC), WHO, International Training and Education Center for Health (I-TECH), International Center for AIDS Care and Treatment Program (ICAP), Population Services International (PSI), ICAP and Doctors Without Borders. There were 4 notification levels of notification. Reporting started at circumcision unit, then data were sent to the district level, to the province and to the central level (MOH). The reports reached the MOH every 30 days and the feedback varied between 60 and 90 days. The system operated using the internet, telephone message and operated both online and offline. The final score obtained for simplicity was 1 point (3/4), corresponding to ≥80%. Although the system involved lot of organizations, according to the evaluation parameters, the system was classified as simple.

**Acceptability:** based on the MOH VMMC target, 10,128 reports were expected to be captured by the system under the period of review. However, only 2,093 (20.7%) were recorded. Of a total of 0.3% (4,652/1,784,335) of individuals who had a VMMC adverse event, all were followed up within 48 hours, on days 7 and 14. The system obtained a score of 1 for acceptability and hence it was considered to have low acceptability (1/2).

**Flexibility:** initially, the system was based in the Integrated Health Information System (HIS) for monitoring and evaluation (basic module) that aggregated and reported routine data through monthly forms completed at the health facility level, which were transmitted monthly via each administrative level to the MOH. In 2013, a more advanced system SIS-MA, was implemented, which had new forms to support the collection, analysis, interpretation and dissemination of health data. The system was able to continue to function with the new changes (e.g. in the introduction of new male circumcision complication reporting forms, new male circumcision follow-up reporting forms). The system had a score of 1 (1/1) point corresponding to 100% and was considered flexible.

**Data quality:** of the 30 essential variables that must be entered, the system had over 80% of the key essential data complete, which corresponds to a score of 4/4 for data quality. Analyzing the completeness of essential variables such as procedures, HIV serological status, follow-up information, and circumcision complication, we found ≥90% of the variables had a value for each observation; the data quality rating has good quality. As for consistency, we calculated the agreement of the data reported by SIS-MA and the data from the circumcision program report, the agreement of the data was ≥90%, and data deviation was 2.0% (Table 2).

**Table 2:** summary of voluntary medical male circumcision (VMMC) procedures in Mozambique, SIS-MA and Report 2013-2019

Year	VMMC procedures performed-SIS-MA	Target	Proportion in (%)	Number of HIV positive individuals circumcised	Number of VMMC adverse events	VMMC procedures performed report	Target	Proportion in (%)
2013	146,046	146,046	100.0	3,221	536	146,046	393,045	37.2
2014	162,670	517,444	31.4	0	650	162,670	517,444	31.4
2015	198,340	450,940	44.0	2,361	561	198,340	450,940	44.0
2016	253,079	279,764	90.5	3,733	233	253,079	279,764	90.5
2017	315,380	178,255	176.9	5,863	567	315,380	178,255	176.9
2018	318,230	156,034	203.9	5,183	2,915	311,891	156,034	199.9
2019	390,590	162,052	241.0	7,683	920	390,590	162,052	241.0
Total	-	2,000,000	-	-	-	-	-	-

**Stability:** this attribute was evaluated using the frequency of interruptions in system functioning due to lack of personnel. We verified that there was no interruption during the period assessed. It had a dedicated person and operational structure for VMMC data entry. The system was classified as stable.

**Opportunity/timeliness:** analyzing the steps and speed of the data collection, transmission, data analysis, and communication of results, the system had a positive evaluation in the 3 parameters analyzed. All reports were available at the stipulated time. The system was classified as timely.

**Figure 2:**
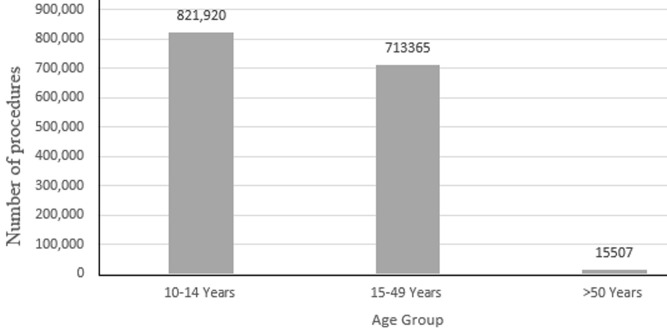
voluntary medical male circumcision (VMMC) by age group in Mozambique, 2013-2019

**Representativeness:** of 1,784,335 VMMC procedures performed during the revised period, the highest proportion 46% (821,920/1,784,335) was performed in the 10-14 age group, followed by the 15-49 age group with 40% (713,365/1,784,335). The age group less frequently circumcised was 50 years or more with 1.9% (15,507/784.335) of the total VMMC procedures (Figure 2). Of the individuals who performed the circumcision, 0.7% (12,391/1,784,335) were positive for HIV/AIDS virus and 0.3% (6382/1,784,335) for adverse events in the period 2013-2019. The largest VMMC coverage was 23.1%, 1 men in 2018 and 28.3% in 2019 (Table 1, Table 1 suite). The year 2019 reported 0.2% (2,915/1,784,335) cases of adverse events of VMMC and Zambézia Province had the highest proportion of male circumcision 22.2% (396,876/1,784,335) while its coverage was low at 16.0% (396,876/2476395) as illustrated in Table 3. The system was considered representative and was able to capture the cases per person, time and place.

**Table 3:** voluntary medical male circumcision (VMMC) procedures and coverage by province in Mozambique, 2013-2019

Indicators	Maputo City	Maputo Province	Tete	Manica	Gaza	Sofala	Zambézia
Procedures	107,104	117,043	160,893	174,357	180,459	222,550	396,876
Coverage (%)	19.7	12.4	12.0	18.0	27.0	20.3	16.0

## Discussion

This evaluation reinforced the importance of the VMMC and the role of SIS-MA in improving the information system in the country, based on the contribution of the findings that can support decision-making by stakeholders in particular for the MOH of Mozambique. Although the number of circumcisions had increased during the period under review, circumcision coverage remained low. This scenario calls attention to the need to strengthen the healthcare system, increase the provision of VMMC services to new healthcare facilities, and ensure the timely recording of all data in the VMMC information system. This study found that men aged 10 and 14 years were more frequently circumcised and similar results were found by a study published in 2019 in Mozambique, which showed that the most frequently circumcised age group were in the 10-29-year-old age groups and that Zambézia Province had the highest male circumcision coverage. Zambézia Province is the second largest population in the country with a total of 2,476,395 men, according to the last census of 2017, with the majority of the population practicing the Islamic religion compared to other parts of the country, since male circumcision is a common practice among Muslims [12,13]. On the other hand, Zambézia Province had a low VMMC coverage of 16.0% (396,876/2476395) compared to Maputo City which had a lower proportion of VMMC and a high coverage of 19.7% (107,104/543,096). This result may be associated with the fact that Zambézia has areas of difficult access to the communities due to the extension of the province and the geographical location.

Despite good data quality, the system recorded a concordance ≥90% between the reporting and SIS-MA data of in 2013. This concordance may be associated with the transition from the basic module to SIS-MA. Routine data quality assessment should be an integral part of a VMMC service quality improvement strategy and facilitate EQA assessments. To maximize and ensure the quality of VMMC services, analyses should be performed to ensure that lessons are put into practice at the site level. Periodic studies to address quality of care should also be put in place (e.g. barriers to services, customer perspectives etc.). The low acceptability may be associated with the failure to send the reports, linked to the deficiency of staff involvement in the information system. Although the rates of VMMC adverse event were low, the program should continue to identify, manage and monitor all possible intra- and post-operative complication as quickly and efficiently as possible. Based on the international scientific recommendation neonatal circumcision carries less risk and confers greater benefits than circumcision later in life [14-17].

This system was considered useful for notifying the complication of circumcision in a timely manner. This rapid notification of complications of VMMC to the national level allowed decision-makers to make timely decisions about the procedure, the same results were found by a study on evaluation of prevention of mother-to-child transmission national health information system for HIV/AIDS, in southern region of Mozambique [18-20].

## Conclusion

The system was representative, flexible, simple, with good data quality and low acceptability. The province has the highest number of procedures but low VMMC coverage in Zambézia Province. We recommended to continue to enter data into the system routinely, guide organizations to better functioning, and extend VMMC services to other areas where circumcision is low. Although HIV/AIDS positivity was lower in men who participated in the study, it is necessary to continue to link prevention measures such as condom use, abstinence, avoidance of early marriage. Increase VMMC coverage in areas with poor access.

**Financing:** the Mozambique Field Epidemiology and Laboratory Training Program (FELTP) was funded through the PEPFAR cooperative agreement NU2GGH002021.

### What is known about this topic

- Voluntary medical male circumcision (VMMC) is one of the crucial strategies for the reduction of the HIV/AIDS in the world and in Mozambique;
- Regular and routine evaluation of health surveillance/information systems is crucial for improving health systems functioning.

### What this study adds

- The VMMC program in Mozambique has made progress in increasing VMMC coverage in priority provinces;
- There was systematic monitoring of the activities carried out by the organizations involved in the health information system in Mozambique;
- There was low coverage in provinces with a higher proportion of VMMC procedures.
